# Identification of Interleukin-27 (IL-27)/IL-27 Receptor Subunit Alpha as a Critical Immune Axis for *In Vivo* HIV Control

**DOI:** 10.1128/JVI.00441-17

**Published:** 2017-07-27

**Authors:** M. Ruiz-Riol, D. Berdnik, A. Llano, B. Mothe, C. Gálvez, S. Pérez-Álvarez, B. Oriol-Tordera, A. Olvera, S. Silva-Arrieta, M. Meulbroek, F. Pujol, J. Coll, J. Martinez-Picado, C. Ganoza, J. Sanchez, G. Gómez, T. Wyss-Coray, C. Brander

**Affiliations:** a IrsiCaixa, AIDS Research Institute, Hospital Germans Trias i Pujol, Autonomous University of Barcelona, Badalona, Spain; b Department of Neurology and Neurological Sciences, Stanford University School of Medicine, Stanford, California, USA; c Fundació Lluita Contra La Sida Hospital Universitari Germans Trias i Pujol, Badalona, Spain; d Projecte dels NOMS-Hispanosida BCN Checkpoint, Barcelona, Spain; e IMPACTA, Lima, Peru; f Universitat Politècnica de Catalunya-Barcelona Tech, Barcelona, Spain; g Institució Catalana de Recerca i Estudis Avançats (ICREA), Barcelona, Spain; h Universitat de Vic–Universitat Central de Catalunya (UVic-UCC), Vic, Spain; University of Southern California

**Keywords:** communicome, HIV, HIV replication control, IL-27 cytokine, Th17 signaling, Wnt signaling, soluble communication factors

## Abstract

Intact and broad immune cell effector functions and specific individual cytokines have been linked to HIV disease outcome, but their relative contribution to HIV control remains unclear. We asked whether the proteome of secreted cytokines and signaling factors in peripheral blood can be used to discover specific pathways critical for host viral control. A custom glass-based microarray, able to measure >600 plasma proteins involved in cell-to-cell communication, was used to measure plasma protein profiles in 96 HIV-infected, treatment-naive individuals with high (>50,000) or low (<10,000 HIV RNA copies/ml) viral loads. Univariate and regression model analysis demonstrate that plasma levels of soluble interleukin-27 (IL-27) are significantly elevated in individuals with high plasma viremia (*P* < 0.0001) and are positively correlated with proviral HIV-DNA copy numbers in peripheral blood mononuclear cells (PBMC) (Rho = 0.4011; *P* = 0.0027). Moreover, soluble IL-27 plasma levels are negatively associated with the breadth and magnitude of the total virus-specific T-cell responses and directly with plasma levels of molecules involved in *Wnt/β-catenin* signaling. In addition to IL-27, gene expression levels of the specific IL-27 receptor (*IL27RA*) in PBMC correlated directly with both plasma viral load (Rho = 0.3531; *P* = 0.0218) and the proviral copy number in the peripheral blood as an indirect measure of partial viral reservoir (Rho = 0.4580; *P* = 0.0030). These results were validated in unrelated cohorts of early infected subjects as well as subjects before and after initiation of antiretroviral treatment, and they identify IL-27 and its specific receptor as a critical immune axis for the antiviral immune response and as robust correlates of viral load and proviral reservoir size in PBMC.

**IMPORTANCE** The detailed knowledge of immune mechanisms that contribute to HIV control is a prerequisite for the design of effective treatment strategies to achieve HIV cure. Cells communicate with each other by secreting signaling proteins, and the blood is a key conduit for transporting such factors. Investigating the communication factors promoting effective immune responses and having potentially antiviral functions against HIV using a novel focused omics approach (“communicome”) has the potential to significantly improve our knowledge of effective host immunity and accelerate the HIV cure agenda. Including 140 subjects with variable viral loads and measuring the plasma levels of >600 soluble proteins, our data highlight the importance of Th17 cells and *Wnt/β-catenin* signaling in HIV control and especially identify the IL-27/IL-27 receptor subunit alpha (IL-27RA) axis as a predictor of plasma viral load and proviral copy number in the peripheral blood. These data may provide important guidance to therapeutic approaches in the HIV cure agenda.

## INTRODUCTION

A small but considerable proportion (1 to 3%) of individuals can control their HIV infection in the absence of antiretroviral treatment (1, 2), while others progress to full-blown AIDS within a few years after infection (3). Despite extensive cohort studies and detailed host genetic studies, proteomics analyses, and host gene expression profiling, the mechanisms that mediate this relative *in vivo* viral control remain poorly understood (4–7). We put a special focus on secreted proteins that are involved in cell-to-cell communication within and across tissues (such as growth factors, cytokines, or chemokines) and for which the blood is a key conduit for their transport. Measuring such soluble plasma “communicome” profiles has served for the prediction of early-onset Alzheimer disease, where testing an overall small cohort for a relatively limited set of soluble plasma factors (*n* = 120) yielded specific profiles predictive of early disease onset (8). Many of these soluble factors are essential in orchestrating an effective host immune response, and some of them may, in the setting of viral infections such as HIV or hepatitis C virus, also possess direct antiviral properties.

Here, we applied the communicome approach to test whether the analysis of communication factors in the peripheral blood of HIV-infected individuals would yield individual cytokines or cytokine profiles predictive of viral control. Our results identify plasma interleukin-27 (IL-27) levels and IL-27 receptor subunit alpha (IL-27RA) expression in peripheral blood mononuclear cells (PBMC) as markers of HIV load and blood viral reservoir size. Moreover, the differential plasma levels of IL-27 are also related, with dysregulated plasma levels of other members of the IL-17 family and several *Wnt/β-catenin* signaling factors, which may explain the well-known imbalance of Th17/T-regulatory (Treg) cells during uncontrolled chronic HIV infection.

## RESULTS

### Communicome profiles associated with HIV chronic untreated infection.

**FIG 1:**
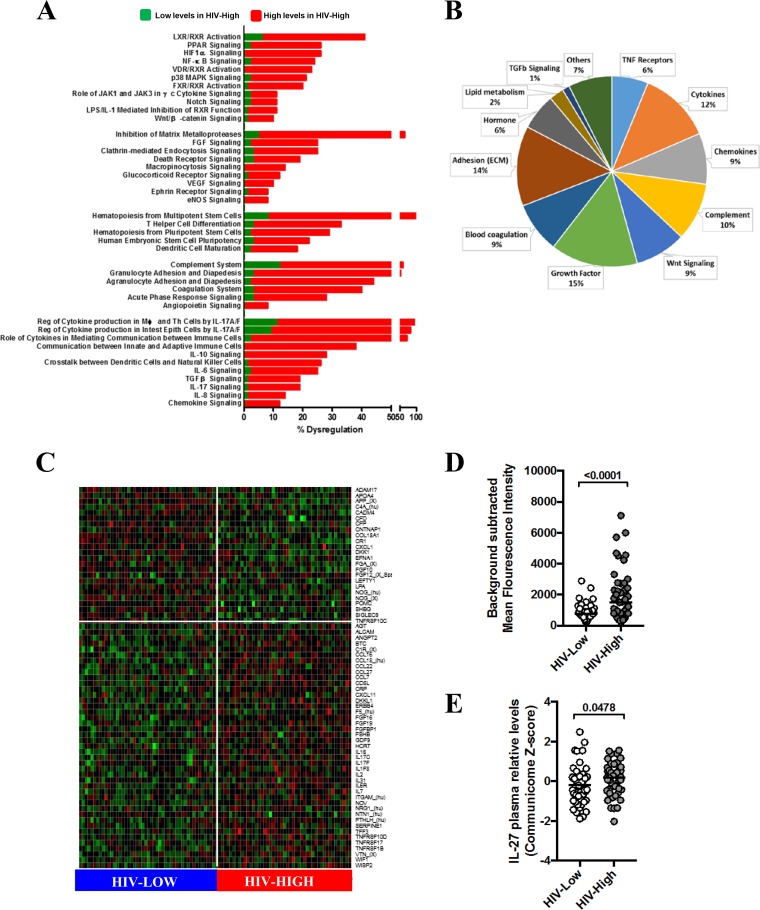
Communicome pattern profiles associated with control of HIV replication. Communicome analysis was performed in 96 HIV-infected individuals with high (HIV-High; *n* = 47) and low (HIV-Low; *n* = 49) HIV plasma viral load in the absence of ART. (A) The percentage of soluble factors with significant differential plasma levels (*P* < 0.05) between HIV-High and HIV-Low is shown for the most severely dysregulated canonical pathways. Elevated and reduced plasma levels of communicome factors in HIV-infected patients with high viral loads are marked in red and green, respectively. PPAR, peroxisome proliferator-activated receptor; MAPK, mitogen-activated protein kinase (MAPK); FGF, fibroblast growth factor; VEGF, vascular endothelial growth factor; eNOS, endothelial nitric oxide synthase; TNF, tumor necrosis factor; FXR, farnesoid X receptor; LXR, liver X receptor; VDR, vitamin D receptor; RXR, retinoid X receptor; ECM, extracellular matrix; MWU, Mann-Whitney U test. (B) Pie charts indicating Gene Ontology Biofunction representation of Top80 factors with significant differences (MWU, *t* test, and false discovery rate of <0.1) between HIV-High and HIV-Low. (C) Heatmap of top 80 soluble factors identified by univariate analyses that provided the most stringent stratification of HIV-High and HIV-Low (red color indicates higher protein plasma levels, and green indicates lower protein plasma levels). (D) IL-27 mean fluorescence intensity levels (5 replicates) detected at 488-nm absorbance in communicome in HIV-Low and HIV-High groups after background subtraction from mean foreground. (E) IL-27 relative expression levels measured in communicome in HIV-Low and HIV-High groups after Z-score normalization of all the chip data. Mann-Whitney test was applied for group comparisons, and *P* values of <0.05 were considered significant.

In order to test whether plasma communicome analysis of HIV-infected individuals with different levels of viremia would yield cytokine profiles predictive of relative viral control *in vivo*, we used custom glass-based microarrays printed with commercially available antibodies to detect a total of 612 secreted signaling proteins (see Table S1 in the supplemental material, communicome chip). This first analysis included plasma samples from HIV-infected patients with low (HIV-Low; *n* = 49) and high (HIV-High; *n* = 47) plasma viral loads (Table S2). Global pathway analysis highlighted several strongly dysregulated immune pathways (Fig. 1A), among them cytokines involved in IL-6, IL-8, IL-10, IL-17, and transforming growth factor beta (TGF-β) signaling. The dysregulated IL-17 signaling was especially strongly supported by the involvement of additional canonical pathways that intersect with IL-17 function and by the presence of several members of the IL-17 cytokine family (IL-17C and IL-17F) among the 80 most differentially expressed factors (Top80) (Fig. 1B and C). Furthermore, HIV-High and HIV-Low patients showed different expression of markers involved in the dendritic cell maturation and global inflammatory process (acute-phase response, complement system, coagulation cascade, and diapedesis) pathways (Fig. 1A to C), possibly reflecting an increased immune activation due to ongoing HIV replication. The communicome data also revealed strongly dysregulated pathways involving inhibition of metalloproteases, death receptors (including TNF receptors family) and Wnt/β-catenin signaling (Fig. 1A to C). Wnt/β-catenin signaling was also prominently represented among the Top80 soluble proteins (9%, including factors DKK1, WNT1, WISP2, and WIF1).

The association between IL-17 and Wnt/β-catenin signaling pathways and the levels of viremia was further validated by multivariate analyses using a recently described approach (forward and all-subsets regression for model selection, or FARMS [9]). FARMS analyses revealed around 40 soluble factors for each round of execution that were strongly associated with HIV load. Table 1 provides the best-fit model for prediction of HIV viremia (Bayesian information criterion [BIC], 103.1283; Akaike information criterion [AIC], −9.702973), where IL-17D (also known as IL-27 and referred to here as such) as well as Wnt inhibitory factor 1 (WIF1) were present in the model. Moreover, correlation analyses indicated that IL-27 was the statistically most robust factor associated with viral load (Rho = 0.3084; *P* = 0.0063; q = 0.0376 by Spearman rank test) among the factors identified by FARMS. Overall, these global analyses indicated that IL-17 signaling (especially IL-27) (Fig. 1D and E) and, to a lesser extent, the Wnt/β-catenin pathway are critical pathways for HIV control during chronic infection.

**TABLE 1:** Forward and all-subsets regression for model selection for viral load

FARMS target	Estimate	SE	*t* value	Pr(>|*t*|)	Significance^*a*^
Intercept	−7.73E+01	2.51E+02	−0.308	0.759395	
ACVR2A	−7.76E−02	5.97E−03	−12.997	<2.E−16	***
BMP2	−6.18E−01	3.59E−02	−17.230	<2.E−16	***
BMP3	1.26E−01	1.78E−02	7.116	2.93E−09	***
CCL13_hu	4.51E−02	3.75E−03	12.022	<2.E−16	***
CCL13_X	−8.38E−02	2.89E−02	−2.906	0.005339	**
CCL20	1.81E−02	5.28E−03	3.434	0.001163	**
CCL21	1.88E−02	9.03E−03	2.079	0.042505	*
CLEC11A	−6.96E−02	4.56E−03	−15.239	<2.E−16	***
CNTNAP1	−5.49E−01	2.84E−02	−19.322	<2.E−16	***
COL18A1	−3.39E−01	2.65E−02	−12.816	<2.E−16	***
CSF1	−1.11E−02	3.56E−03	−3.133	0.002820	**
CST3	−1.07E−02	3.47E−03	−3.079	0.003287	**
CTF1	−3.50E−02	8.50E−03	−4.111	0.000138	***
CXCL16	5.31E−01	3.73E−02	14.218	<2.E−16	***
FGF17	4.23E−02	4.06E−03	10.421	1.95E−14	***
FGF3_hu	2.22E−01	4.81E−02	4.617	2.52E−05	***
FTL_hu	−2.75E−02	6.59E−03	−4.176	0.000111	***
GRN_X	3.59E−01	3.02E−02	11.894	<2.E−16	***
HCRT	6.85E−02	4.42E−03	15.497	<2.E−16	***
ICAM2	2.76E−02	3.57E−03	7.732	3.00E−10	***
IL-12A	6.05E−02	1.99E−02	3.041	0.003665	**
IL-17D	−2.49E−02	5.40E−03	−4.615	2.54E−05	***
LTA	6.29E+00	3.26E−01	19.289	<2.E−16	***
MMP25	−5.85E−01	9.19E−02	−6.367	4.69E−08	***
NGFR	−8.52E−03	4.98E−03	−1.710	0.093029	·
NLGN1_hu	−4.77E−01	2.24E−01	−2.125	0.038236	*
NLGN1_X	−1.19E−01	4.10E−02	−2.896	0.005486	**
NLGN4X	1.26E−01	2.50E−02	5.050	5.58E−06	***
NOG_hu	−9.37E−01	4.56E−02	−20.552	<2.E−16	***
NOG_X	−4.50E−01	1.34E−01	−3.356	0.001469	**
NPY_X	3.47E+00	2.24E−01	15.507	<2.E−16	***
NRG1_hu	7.93E−02	8.80E−03	9.013	2.80E−12	***
SERPING1	1.02E−01	8.17E−03	12.533	<2.E−16	***
SIGIRR	3.24E−02	4.02E−03	8.070	8.65E−11	***
SLC2A1	−2.15E−01	2.08E−02	−10.373	2.30E−14	***
SORL1_hu	1.08E+00	1.86E−01	5.796	3.82E−07	***
SPARC_hu1	−5.85E−02	8.72E−03	−6.712	1.31E−08	***
SPARC_hu2	−5.92E−01	8.40E−02	−7.044	3.84E−09	***
THBS4	2.05E−01	3.93E−02	5.226	2.98E−06	***
TIE1	−6.15E−02	8.83E−03	−6.960	5.22E−09	***
VEGFC	−1.39E+00	9.69E−02	−14.374	<2.E−16	***
WIF1	1.74E−01	1.44E−02	12.070	<2.E−16	***

a Significance codes: ***, <0.001; **, <0.01; *, <0.05; ·, <0.1. Residual standard errors, 0.1958 on 53 degrees of freedom; multiple *R*^2^, 0.9897; adjusted *R*^2^, 0.9815; F statistic, 120.9 on 42 and 53 degrees of freedom; *P* < 2.2E−16. Residuals: minimum, −0.43703; 1st quartile, −0.08250; median, −0.00196; 3rd quartile, 0.07220; maximum, 0.52013. AIC, −9.702973; BIC, 103.1283; Pr(>|*t*|), proportion of *t* distribution greater than *t* statistic.

### IL-27 plasma levels are associated with viremia and reduced HIV-specific T-cell responses.

**FIG 2:**
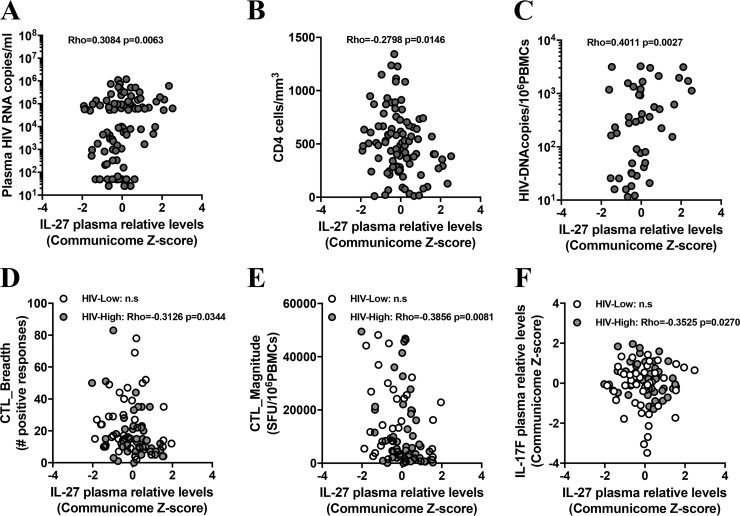
IL-27 plasma levels during HIV chronic infection are associated with viral load, reservoir size, HIV-specific CTL responses, and IL-17F plasma levels. Relative IL-27 plasma levels (*x* axis) are compared to either plasma HIV RNA copies per milliliter of peripheral blood (A), CD4 cell counts in the peripheral blood (B), proviral DNA levels per million of PBMC (C), the breath (D) and the magnitude (E) of T-cell responses against the entire HIV proteome detected by IFN-γ ELISpot assay, or IL-17F relative plasma levels (F). All 96 subjects are represented (dark gray circles), and HIV-High (light gray circles) and HIV-Low (open circles) individuals are shown. Spearman rank test for correlation studies was applied, and associations were considered significant for *P* values of <0.05. n.s., not significant.

Given the described effects of the IL-17 cytokine family on immune function, and IL-27 in particular, on HIV infection (10–12), we asked how plasma levels of IL-17A, IL-17B, IL-17C, IL-17F, and IL-27 were related to HIV load and CD4^+^ T-cell counts. As shown in Table 2 and Fig. 2A and B, plasma levels of IL-27 showed the strongest significant correlations with these 2 parameters (for viral load, Rho of 0.3084 and *P* value of 0.0063; for CD4, Rho of −0.2798 and *P* value of 0.0146; Spearman rank test) among the tested IL-17 cytokines. In order to study the relationship between relative plasma levels of IL-17 cytokines and latent reservoir in peripheral blood, proviral DNA levels in total PBMC were measured by droplet digital PCR (ddPCR). Of note, since latent reservoir (persistently infected cells) can be detected in blood but are also distributed in different tissues (such as gastrointestinal tract, genital tract, and central nervous system) (13), and since the ddPCR approach detects both integrated virus and productively infected cells (14), measuring HIV proviral DNA copy numbers in PBMC reflects only a partial latent reservoir. Still, strong associations were observed between IL-27 plasma levels and proviral DNA levels in total PBMC (Rho = 0.4011; *P* = 0.0027; Spearman rank test) (Fig. 2C), suggesting an important role of this cytokine in the establishment and/or maintenance of a latent reservoir, at least in the peripheral blood.

**TABLE 2:** Plasma protein levels of IL-17 family members measured in communicome associated with clinical and virological parameters

IL-17 member	Viral load (plasma HIV RNA copies/ml)		CD4 count (cells/mm^3^)		Proviral load (HIV-DNA copies/10^6^ PBMCs)	
	Rho	*P* value	Rho	*P* value	Rho	*P* value
IL-17A	0.2398	0.0186	−0.1290	0.2105	0.0905	0.5112
IL-17B	0.2119	0.0382	−0.1270	0.2175	0.1502	0.2736
IL-17C	0.2871	0.0046	−0.2332	0.0222	0.1452	0.2900
IL-17D (IL-27)	0.3084	0.0063	−0.2798	0.0146	0.4011	0.0027
IL-17F	0.1594	0.1209	−0.1744	0.0893	0.0736	0.5933

Among the multiple proinflammatory effects that IL-27 exerts *in vivo* is the promotion of T-cell proliferation, production of gamma interferon (IFN-γ) by CD4^+^ T cells, and the driving of strong cytotoxic T lymphocyte (CTL) responses (10, 12). Thus, the relationship between the plasma levels of IL-27 and the *ex vivo* CTL responses against the entire HIV proteome was assessed. This analysis showed an inverse correlation between IL-27 levels and the total CTL breadth (measured by IFN-γ enzyme-linked immunosorbent spot [ELISpot] assay; Rho = −0.3126; *P* = 0.0344; Spearman rank test) and the total CTL magnitude (Rho = −0.3859; *P* = 0.0081; Spearman rank test) in HIV-infected patients with high viral loads (Fig. 2D and E), indicating that the higher plasma levels of IL-27 were associated with weaker CTL responses in this group. On the other hand, IL-27 has well-described immune-regulatory properties, among them the ability to repress Th17 responses, which may lead to reduced frequencies of Th17 cells in HIV-infected subjects with high plasma viremia. Indeed, the HIV-High individuals, who showed higher levels of IL-27 in the plasma than the HIV-Low group (Fig. 2F), also showed an inverse correlation between IL-27 and IL-17F (one of the hallmark cytokines of Th17 cells) (Rho = −0.3525; *P* = 0.0270; Spearman rank test). No statistically significant association between IL-27 or IL-17F levels was observed in HIV-Low subjects (Fig. 2F). Overall, these data suggest that the IL-17 cytokine family, particularly IL-27, have a profound impact on HIV reservoir in peripheral blood, viral replication, CTL responses, and HIV disease progression, and that elevated levels of IL-27 are linked to uncontrolled viral replication and weaker HIV-specific immune responses.

### *Wnt/β-catenin* signaling activation is associated with IL-27 plasma levels in chronic uncontrolled HIV infection.

**FIG 3:**
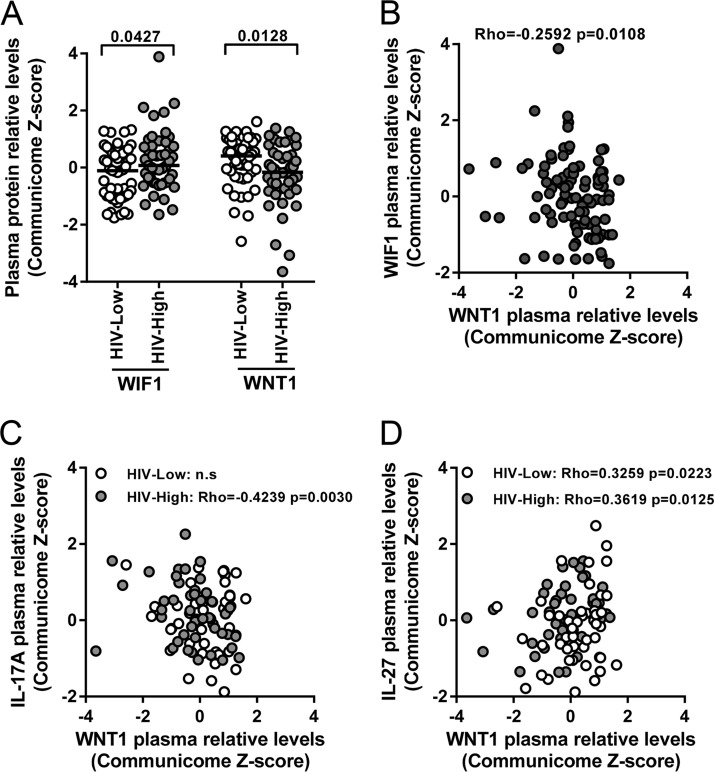
Plasma levels of Wnt/β-catenin pathway proteins are associated with IL-17 signaling in chronic HIV infection. (A) Differential relative plasma levels of Wnt/β-catenin inhibitor 1 (WIF1) and activator (WNT1) factors between HIV-High (light gray circles) and HIV-Low (open circles) detected in the communicome screens. (B) Inverse relationship between relative plasma levels of WNT1 (*x* axis) and WIF1 (*y* axis) across all individuals. (C) Inverse correlation between relative plasma levels of WNT1 (*x* axis) and IL-17A (*y* axis) in HIV-High (light gray circles) and HIV-Low (open circles) subjects. (D) Positive association between relative plasma levels of WNT1 (*x* axis) and IL-27 (*y* axis) detected in HIV-High (light gray circles) and HIV-Low (open circles) subjects. The Mann-Whitney test was applied for group comparisons and the Spearman rank test for correlation analyses. *P* values of <0.05 were considered significant.

The *Wnt/β-catenin* pathway is centrally involved in major physiologic cellular functions, such as proliferation, migration, and pluripotency maintenance. During recent years, a direct relationship between *Wnt/β-catenin* signaling factors and control of viral infections, including cytomegalovirus and HIV infection, has emerged (15–18). In line with these data, we found plasma levels of several factors involved in the *Wnt/β-catenin* pathway (including activators [e.g., WNT1] as well as inhibitors [e.g., WIF1]) to be differentially expressed between HIV-Low and HIV-High individuals (Fig. 3A and B). In addition, the *Wnt/β-catenin* activating factor WNT1 was inversely associated with viral load (Rho = −0.2775; *P* = 0.0062; Spearman rank test; data not shown) and also correlated inversely with IL-17A in patients with high plasma viremia (Rho = −0.4239; *P* = 0.0030; Spearman rank test) (Fig. 3C). These data suggest that reduced levels of IL-17A (indirect measure of Th17 responses) in HIV-High subjects are under *Wnt/β-catenin* regulation. This is further corroborated by our finding that plasma levels of IL-27 and WNT1 were directly correlated in both HIV-Low (Rho = 0.3259; *P* = 0.0223; Spearman rank test) (Fig. 3D) and in HIV-High groups (Rho = 0.3619; *P* = 0.0125; Spearman rank test) (Fig. 3D). These observations are in line with recent reports showing that *Wnt/β-catenin* signaling promotes the expression of IL-27 in dendritic cells and that secreted IL-27 suppresses Th17 responses (19). Taken together, these communicome data establish a relationship between the *Wnt/β-catenin* and IL-17 family signaling pathways with the *in vivo* control of HIV, with IL-27 potentially acting as the interconnecting factor between these pathways.

### Lower plasma levels of IL-27 are linked to reduced *IL27RA* expression in PBMC of HIV controllers.

**FIG 4:**
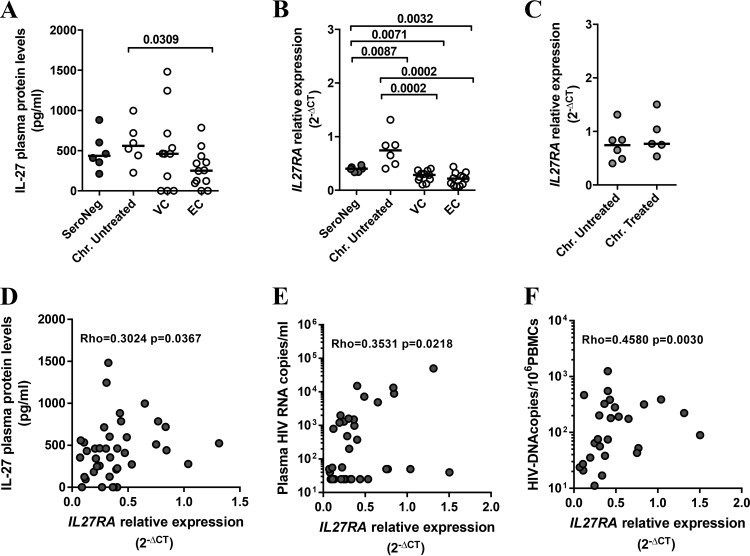
Low IL-27/IL27RA plasma levels during HIV chronic infection are associated with viral control. (A) Differential plasma levels of IL-27 detected by ELISA (picograms/milliliter) are shown for samples from validation cohorts, including HIV seronegatives (SeroNeg) (*n* = 6), chronically infected, viremic, untreated HIV-infected individuals (Chr. Untreated) (*n* = 6), HIV viremic controller subjects (VC; *n* = 11), and HIV elite controller patients (EC; *n* = 12). (B) HIV seronegatives (*n* = 6), chronically infected, viremic, untreated HIV-infected individuals (*n* = 6), HIV viremic controller subjects (VC; *n* = 11), and HIV elite controller patients (EC; *n* = 12). (C) Relative expression levels of *IL27RA* detected in PBMC by RT-PCR in chronically viremic infected subjects. Untreated, *n* = 6; treated, *n* = 5. (D to F) Correlation between *IL27RA* gene expression in PBMC (*x* axis) and plasma levels of IL-27 (*y* axis) (D), plasma viral loads (E), and proviral DNA copy number in total PBMC (F) in validation cohorts. The Mann-Whitney test was applied for groups comparisons and Spearman rank test for correlation studies. *P* values of <0.05 were considered significant.

To validate the signals emerging from the communicome analyses in the above-described 96 individuals, we assessed plasma levels of IL-17 family cytokines in unrelated cohorts, including seronegative subjects, chronically infected individuals (before and after combined antiretroviral treatment [cART] initiation), and controller subjects (including viremic [VC] and elite [EC] HIV controllers) (Fig. 4; Table S3 and Fig. S1). Although the group sizes were considerably smaller than those in the initial cohort, significantly lower IL-27 plasma levels were seen in elite controller individuals compared to chronically untreated infected subjects with high viremia (Fig. 4A), confirming the data obtained in the communicome analyses. Longitudinal analyses of individuals who were tested a median of 12 months before and after initiation of cART did not show any significant reduction of IL-27 plasma levels upon treatment (data not shown). This suggests that reducing viremia by cART does not readily restore a low IL-27 profile, at least not within 12 months on suppressive treatment. No differences in IL-17A and IL-17F plasma levels were detected between the various groups (Fig. S1), suggesting that among the different IL-17 family members, IL-27 plays the most prominent role in the outcome of HIV infection.

IL-27 binds to its heterodimeric receptor, composed of the IL-27 receptor α chain (IL-27RA) and the gp130 chain, which is shared with other heterodimeric receptors for other cytokines, particularly IL-6 (10). Since IL-27RA mediates specificity to IL-27 binding, the expression level of this subunit was measured in RNA isolated from total PBMC from HIV-seronegative individuals, chronically infected subjects (with and without cART), and controllers. Compared to HIV-uninfected subjects, HIV-infected individuals with uncontrolled and untreated HIV infection showed an increased expression of *IL27RA* (Fig. 4B), while in HIV controllers (viremic and elite controllers) its expression was low and at levels seen in HIV-seronegative individuals (Fig. 4B). Of note, among HIV controllers, viremic subjects (<2,000 copies/ml) and elite controllers (<50 copies/ml) showed equally low *IL27RA* expression (Fig. 4B). Similarly, separating viremic noncontrollers into groups that did or did not receive treatment did not reveal any differences in expression of *IL27RA* (Fig. 4C). More importantly, *IL27RA* expression levels across all groups (controllers and noncontrollers as well as HIV-negative individuals) were directly correlated with the concentration of IL-27 cytokine in the plasma (Rho = 0.3024; *P* = 0.0367; Spearman rank test) (Fig. 4D), establishing a link between coordinated IL-27 cytokine, *IL27RA* expression, and HIV control. Accordingly, and as seen with IL-27, *IL27RA* also positively correlated with viral load (Rho = 0.3531; *P* = 0.0218; Spearman rank test) and proviral DNA levels in total PBMC (Rho = 0.4580; *P* = 0.0017; Spearman rank test) (Fig. 4E and F). Taken together, these data indicate that reduced levels of IL-27 and *IL27RA* serve as markers of HIV load and proviral DNA levels in total PBMC.

## DISCUSSION

A better understanding of the mechanisms by which host immunity contributes to HIV control in the absence of antiretroviral treatment will be needed to guide future immune-based interventions, especially in the therapeutic vaccination setting. To date, the fine specificity of HIV-specific CD8^+^ T cells, the maintenance of their polyfunctionality, and the ability of virus-specific CD8^+^ T cells to suppress *in vitro* viral replication has been most consistently associated with reduced viral loads in chronic HIV infection (20–23). Here, we applied, for the first time, an innovative communicome approach that was initially utilized to predict early onset of Alzheimer disease (8) to identify potential soluble biomarkers involved in cellular communication that are associated with HIV control. Our data show that elevated levels of IL-27 are associated with higher viral load and increased size of the viral reservoir, and that these effects may be mediated by a dysregulated *Wnt/β-catenin* signaling pathway.

A particularity of the present study was the inclusion of individuals with high or low viral loads in the initial communicome screens rather than focusing on elite or low-viremia controllers. This allowed us to avoid bias toward host genetics, which are often skewed in controller cohorts, and to identify signals that are reflective of a larger proportion of the HIV-infected population than the small subset of (elite) controllers (24, 25). Still, the subsequent replication of the observed results in unrelated cohorts, including elite controllers, validated the chosen approach and identify IL-27 and its receptor *IL27RA* as possibly critical factors in host defense and immunopathology of HIV infection.

IL-27 is a highly pleiotropic cytokine with immune-regulatory and antiviral capabilities. It belongs to the IL-6/IL-12 cytokine family (10, 11) and is composed of the Epstein-Barr virus-induced gene 3 (Ebi3) product and the IL-27p28 chain. Its receptor is composed of gp130 and the IL-27 receptor α chain (IL-27RA; also known as WSX1 or TCCR). From its discovery, IL-27 was seen as a proinflammatory cytokine due to its ability to suppress both Th2 responses and Foxp3^+^ Treg differentiation, expanding Th1 cells, and promoting T-cell proliferation and CTL responses (10, 12). Our data do not support such an effect stimulating T-cell responses, as we observed an inverse association between the plasma levels of IL-27 and the magnitude and breadth of HIV-specific CTL responses, at least in those subjects with high viral loads. It thus appears that at least in this context, IL-27 does not promote the expansion of functional, HIV-specific CTL responses.

Since IL-27 also exerts immunosuppressive effects by inhibiting the development of Th17 cells and by inducing IL-10-producing Tr1 (Treg1 cells) (12), we also assessed the association of IL-27 plasma levels with IL-17A and IL-17F. The inverse correlation between IL-27 and IL-17F observed here and the reportedly higher percentages of Th17 cells in peripheral blood of HIV elite controllers suggest that IL-27 acts as a key repressor of Th1/Th17-like responses in uncontrolled HIV infection (26, 27). Evidently, determining cytokine plasma levels is an indirect measure of Th17 responses, and our data interpretation is further complicated by the pleiotropic effects of IL-27 and its use of shared coreceptors, e.g., the gp130/IL6ST molecule (28). Nevertheless, as uncontrolled HIV infection is characterized by low levels of Th17 cells, our data are compatible with a scenario where low levels of IL-27 and its receptor favor increased Th17 activity and improved viral control (29).

Our data also indicate an involvement of the *Wnt/β-catenin* pathway in the observed association between low IL-27 and relative viral control. This is in line with data that show the *Wnt/β-catenin* pathway is essential for normal T-cell development and that IL-27 suppresses Th17 differentiation (19). As such, IL-27 could impact Treg differentiation by acting on the *Wnt/β-catenin* pathway, thereby contributing to the well-known imbalance between Tregs and Th17 cells during HIV disease progression (26, 27, 30). However, in *in vitro* assays, IL-27 has also been linked with antiviral effects (11, 31), since the addition of exogenous IL-27 to primary cell cultures has been shown to decrease HIV-1 replication in a number of cell types, including PBMC, CD4^+^ T cells, macrophages, and dendritic cells. As we observed a positive association between IL-27/*IL27RA* expression with plasma viral load and, even more pronounced, with proviral DNA levels, our data suggest that the *in vitro* analyses do not fully replicate the situation *in vivo*. One possibility that accounts for this inconsistency is that IL-27 secretion and absorption mechanisms are not the same in the peripheral blood and plasma from HIV-infected patients as those produced during *in vitro* experiments using primary cell cultures from healthy donors in the presence of exogenous IL-27. Moreover, IL-27 plasma levels are a reflection of different cell sources and tissues where other cytokines coexist, including some that share the gp130 subunit of the IL-27 receptor (such as IL-6, IL-11, oncostatin M [OSM], ciliary neurotrophic factor [CNTF], cardiotrophin-1 [CT-1], and leukemia inhibitory factor [LIF]) that may compete for binding with this receptor subunit, as described, for instance, for IL-6 *trans*-signaling (28, 32). In particular, the effects of dysregulated *Wnt/β-catenin* signaling on the self-renewal of CD4^+^ T-cell populations and maintenance of HIV reservoir by homoeostatic proliferation will need to be assessed in the context of IL-27/IL-27RA expression profiles (16–18, 33, 34).

Overall, the application of a communicome approach to plasma samples from HIV-infected individuals demonstrates the potential of such focused proteomics analyses for the identification of new molecules associated with control/noncontrol of HIV infection. Importantly, however, our data also add to a better understanding of immune-regulatory alterations in this highly immunogenic, persistent viral infection. The observation that both IL-27 receptor and ligand were differentially associated with viral load and proviral levels in PBMC also highlights the need for future therapeutic intervention strategies to carefully evaluate whether strategies such as immune checkpoint inhibitors need to be directed against more than a single target molecule.

## MATERIALS AND METHODS

### Patients and samples.

HIV-seropositive subjects (*n* = 96) were recruited at the Hospital Germans Trias i Pujol, (Badalona, Spain) and the IMPACTA clinics in Lima (Peru) and used for communicome analyses. HIV low-viremia subjects (HIV-Low; *n* = 49) were defined as individuals with viral loads of <10,000 HIV RNA copies/ml (range, 25 to 9,990; median, 530) in the absence of antiretroviral treatment; HIV high-viremia individuals (HIV-High; *n* = 47) were untreated subjects with >50,000 HIV RNA copies/ml (range, 50,295 to 1,200,000; median, 105,520) (see Table S2 in the supplemental material). CD4 counts in the HIV-Low groups ranged from 289 to 1,343 cells/mm^3^ (median, 657 cells/mm^3^) and in HIV-High group from 11 to 726 cells/mm^3^ (median, 283 cells/mm^3^) (*P* < 0.0001 by Mann-Whitney test). Additional unrelated cohorts were used for downstream validation of identified signals, including seronegative (*n* = 8) subjects, individuals with samples obtained in the chronic phase of HIV infection (*n* = 12), and subjects sampled pre-cART administration (untreated; median of 70 days before treatment initiation; *n* = 6) and post-cART initiation (treated; median of 362 days after treatment start; *n* = 6) (Table S3). In addition, samples from a chronically infected controller cohort (C; *n* = 23), including viremic (VC; *n* = 11; viral loads of <2,000 copies/ml) and elite controllers (EC; *n* = 12; undetectable viral loads; <50 copies/ml), were included (Table S3). The study was approved by the Comitè Ètic d'Investigació Clínica of Hospital Germans Trias i Pujol (CEIC EO-12-042), and all participants provided written informed consent. Plasma samples were used and processed for proteomic studies (communicome and enzyme-linked immunosorbent assay [ELISA] determinations) as described below. For molecular assays, RNA and DNA were extracted from PBMC.

### Communicome analyses.

Communicome analyses were carried out using a custom-designed chip that allowed for the detection and quantification of >600 individual proteins (Table S1). Multiple specific monoclonal or polyclonal antibodies for each protein were printed in triplicate on SuperEpoxy glass slides using a custom-built robotic microarrayer fitted with 16 SMP4B pins as previously described (8). The slides were dried overnight, vacuum sealed, and stored at −20°C until use. Plasma samples were platelet and lipid reduced by centrifugation and dialyzed (96-well DispoDialyzer with a 5-kDa molecular size cutoff) at 4°C to yield a maximally pure plasma protein fraction. Plasma proteins were subsequently N-terminally biotinylated, and the individual samples were incubated overnight with blocked antibody arrays at 4°C. Finally, the arrays were washed extensively and the captured proteins detected with streptavidin-Alexa 555 on an InnoScan 700 scanner. For data analysis, raw data were background subtracted and normalized using Cluster analysis, and the normalized values were Z scored [*z* = (*x* − μ)/σ]. Differences in the set of samples were analyzed by applying *t* test, Mann Whitney test, and the SAM (Significance Analysis Microarrays) 3.00 algorithm, which assigns a score to each molecule on the basis of multicomparison analyses of expression changes and indicates significance by q value. Molecules with a significance level above 95% (*P* value of <0.05) and a conservative false discovery rate below 10% (q value of <0.1) were included in further analyses.

### Network and array analyses.

The analysis of molecule interactions was performed using Ingenuity Pathway Analysis software (IPA; Ingenuity Systems; www.ingenuity.com), highlighting networks and canonical pathways differentially affected by high and low HIV plasma viremia. Protein-protein interactions and pathway analyses were further supported by including Kyoto Encyclopedia of Genes and Genomes (KEGG; www.genome.ad.jp/kegg), BioCarta, GeneMania (www.genemania.org/), and PubMed literature searches. Heatmaps and hierarchical clustering were performed using Bioconductor software and statistical programming language R (www.r-project.org) (35, 36).

### Cytokine determination by ELISA.

Legend Max human ELISA kits (BioLegend) were used for the quantification of IL-27 (IL-17D), IL-17A, and IL-17F in plasma samples by following the manufacturer's procedure. In brief, after antibody coating, plates were washed and standards (duplicates) and samples (triplicates) were added and incubated during 2 h while shaking. After subsequent washing, human cytokine detection antibodies were added and incubated for 1 h while shaking. Avidin-horseradish peroxidase solution and substrate solution were then sequentially added and incubated for 30 and 15 min, respectively. Finally, stop solution was added and the absorbance at 450 nm determined (absorbance at 570 nm is also measured for background subtraction). For cytokine quantification, a 4-parameter logistic nonlinear regression model was applied, and the absorbance median of 3 replicates was used for quantification.

### Measurements of cytotoxic host immune responses to HIV.

T-cell immunity to HIV was assessed in purified PBMC by ELISpot assay (1 × 10^6^ PBMC/well) using an overlapping peptide (OLP) set of 410 OLP as described previously (37). In brief, 96-well ELISpot plates (Millipore) were coated with mouse anti-human IFN-γ antibody 1-D1k (Mabtech) and incubated overnight. The next day, plates were washed six times with phosphate-buffered saline (PBS), and PBMC were added at 100,000 cells/well along with peptide at a final concentration of 2 μg/ml. After incubation for 16 h at 37°C in 5% CO_2_, plates were washed 6 times with PBS and the mouse anti-human IFN-γ-biotinylated 7-B6-1 (Mabtech) was added for 1 h at room temperature. After washing, streptavidin alkaline phosphatase (Mabtech) was added for 1 h at room temperature. Spots were visualized by adding 5-bromo-4-chloro-3-indolylphosphate and nitroblue tetrazolium (Bio-Rad) for 5 to 10 min. Culture medium alone and phytohemagglutinin P (PHA-P) (10 μg/ml) served as negative and positive controls, respectively. Spots were counted on an automated CTL ELISpot assay reader. The breadth (number of reactive OLP) and total magnitude (median number of spot-forming cells [SFC] per 10^6^ PBMC for all positive responses) were recorded.

### Total HIV-1 DNA quantification.

Total HIV-1 DNA was quantified in PBMC lysates by droplet digital PCR (ddPCR) in duplicate as described previously (38). Briefly, two different primer/probe sets annealing to the 5′ long terminal repeat and Gag regions, respectively, were used to circumvent sequence mismatch in the patients' proviruses, and the RPP30 housekeeping gene was quantified in parallel to normalize sample input. Raw ddPCR data were analyzed using the QX100 droplet reader and QuantaSoft v.1.6 software (Bio-Rad).

### Real-time PCR.

Frozen PBMC were thawed and cell pellets conserved in RNAprotect cell reagent (Qiagen) for RNA extraction (RNeasy Plus minikit; Qiagen) and retrotranscription (SuperScript III first-strand synthesis supermix). The cDNA was used for reverse transcription-PCR using TaqMan gene expression assays for detection of *IL27RA* (Hs00945029_m1) and *TBP* (Hs99999910_m1), all from Applied Biosystems, with a LightCycler (Roche). The relative expression was calculated as 2^−Δ*CT*^ (where *C_T_* is the median threshold cycle from 3 replicates).

### Statistical analysis.

Univariate analyses were performed using GraphPad Prism, version 5. For comparisons between patient groups, nonparametric Mann-Whitney and Wilcoxon matched-pair tests were applied as indicated. Spearman test was applied for correlation analyses. For all analyses, *P* values of <0.05 were considered statistically significant. Forward and all-subsets regression for model selection (FARMS) analysis was applied to define a predictive model for viral load. FARMS is a novel approach initially designed to identify the impact of HLA genetic diversity on HIV loads in extensive cohorts of HIV-infected individuals (9). The method combines forward and all-subsets regression for model selection and can be user adjusted for most input variables and output parameters. FARMS is generally faster than other comparable, commonly used approaches and requires less computational infrastructure (9).

## Supplementary Material

Supplemental material
