# Supplementary material for: Identification of Interleukin-27 (IL-27)/IL-27 Receptor Subunit Alpha as a Critical Immune Axis for In Vivo HIV Control
Source: J Virol. 2017 Jul 27;91(16):e00441-17. doi: 10.1128/JVI.00441-17 (PMC5533920; doi:10.1128/JVI.00441-17)
Supplement: Supplemental material [file supp_91_16_e00441-17__index.html]

Supplemental material 

# Identification of Interleukin-27 (IL-27)/IL-27 Receptor Subunit Alpha as a Critical Immune Axis for *In Vivo* HIV Control

## Supplemental material

- Supplemental file 1 -

  Table S1 (The 612 measured proteins in alphabetic order.)

  Table S2 (Characteristics of HIV-infected patients used for communicome analysis.)

  Table S3 (Characteristics of independent cohorts used for ELISA and RT-PCR validation.)

  Fig. S1 (IL-17A/F plasma levels during HIV infection.)

  PDF, 84K
