# Supplementary material for: Identification of Interleukin-27 (IL-27)/IL-27 Receptor Subunit Alpha as a Critical Immune Axis for In Vivo HIV Control
Source: J Virol. 2017 Jul 27;91(16):e00441-17. doi: 10.1128/JVI.00441-17 (PMC5533920; doi:10.1128/JVI.00441-17)
Supplement: Supplemental material [file JVI.00441-17_zjv999182826s1.pdf]

Table S1: The 612 measured proteins in alphabetic order

Gene	Ab	Gene	Ab	Gene	Ab
**A2M**	alpha-macroglobulin mAb	**BMP7**	BMP-7	**CCL23**	CCL23/CKBeta 8-1 mAb
**ACAN**	Proteoglycan 1 mAb	**BMP8B**	BMP8b mAb	**CCL24**	Eotaxin-2 / MPIF-2
**ACE**	ACE-2	**BMPER**	CV-2 / Crossveinless-2	**CCL25**	TECK / CCL25
**ACVR1**	Activin RIA / ALK-2	**BMPR1A**	BMPR-IA / ALK-3	**CCL26**	Eotaxin-3 / CCL26
**ACVR1B**	Activin RIB / ALK-4	**BMPR1B**	BMPR-IB / ALK-6	**CCL27**	CTACK / CCL27
**ACVR2A**	Activin RIIA	**BMPR2**	BMPR-II	**CCL28**	CCL28 / VIC
**ADAM17**	TACE	**BTC**	BTC	**CCL3**	MIP-1a
**ADIPOQ**	Adiponectin / Acrp30	**C1QA**	C1q	**CCL3L1**	LD78beta pAb
**AFP**	alpha-fetoprotein mAb	**C1S**	C1s	**CCL4**	MIP-1b
**AGRP**	AGRP pAb	**C2**	C2	**CCL5**	RANTES
**AGT**	Angiotensinogen	**C3**	C3a des Arg	**CCL7**	MCP-3
**ALCAM**	ALCAM	**C3**	C3c	**CCL8**	MCP-2
**AMH**	MIS/AMH Propeptide mAb	**C3**	C3d (neoantigen)	**CCR1**	CCR1
**ANG**	Angiogenin	**C3AR1**	C3aR	**CCR2**	CCR2
**ANGPT1**	Angiopoietin-1	**C4A**	C4 Binding Protein	**CCR3**	CCR3
**ANGPT2**	Angiopoietin-2	**C4A**	C4d (neoantigen)	**CCR4**	CCR4
**ANGPT4**	Angiopoietin-4	**C4A**	C4c	**CCR5**	CCR5
**ANGPTL1**	ANGPTL1	**C5**	SC5b-9	**CCR6**	CCR6
**ANGPTL2**	Angiopoietin-like 2	**C5**	rC5a	**CCR7**	CCR7
**ANGPTL3**	ANGPTL3	**C5**	C5	**CCR8**	CCR8
**ANGPTL4**	ANGPTL4	**C5**	C5b neo-epitope	**CCR9**	CCR9
**ANGPTL7**	Angiopoietin-like Factor	**C6**	C6	**CD14**	CD14
**APLNR**	Apelin Receptor	**C7**	C7	**CD163**	CD 163
**APOB**	ApoB	**C8A**	C8	**CD22**	Siglec-2/CD22 mAb
**APOE**	ApoE	**C9**	C9	**CD27**	CD27 / TNFRSF7
**APP**	APP 444-592 mAb (Clone 1)	**CARTPT**	CART	**CD36**	CD36
**AREG**	AR (Amphiregulin)	**CCBP2**	D6	**CD40**	CD40 / TNFRSF5
**ARTN**	Artemin	**CCL1**	I-309	**CD40LG**	TNFSF5 /CD154
**AXL**	Axl	**CCL11**	Eotaxin / CCL11	**CD46**	MCP (CD46)
**B2M**	beta 2 microglobulin mAb	**CCL13**	MCP-4 / CCL13	**CD55**	DAF (CD55)
**BDNF**	Pro-BDNF mAb	**CCL14**	CCL14 / HCC-1 / HCC-3	**CD59**	CD59 (MEM43 epitope)
**BDNF**	BDNF mAb (Clone 1)	**CCL15**	MIP-1d	**CD80**	B7-1 /CD80
**BGLAP**	Osteocalcin	**CCL16**	HCC-4 / CCL16	**CDH5**	VE-Cadherin
**BMP10**	BMP-10 propeptide pAb	**CCL17**	Tarc	**CER1**	Cerberus 1
**BMP15**	BMP-15	**CCL18**	PARC / CCL18	**CFB**	Factor Ba
**BMP2**	BMP-2	**CCL19**	MIP-3 beta	**CFB**	Factor Bb (neoantigen)
**BMP3**	BMP-3	**CCL2**	MCP-1	**CFC1**	Cryptic
**BMP4**	BMP-4	**CCL20**	MIP-3 alpha	**CFD**	Factor D
**BMP5**	BMP-5	**CCL21**	6Ckine	**CFH**	Factor H
**BMP6**	BMP-6	**CCL22**	MDC	**CFI**	iC3b (neoantigen)
**CFI**	Factor I	**CXCR3**	CXCR3	**FGF16**	FGF-16
**CFP**	Factor P	**CXCR4**	CXCR4 (fusin)	**FGF17**	FGF-17
**CHRD**	Chordin pAb	**CXCR5**	CXCR5 /BLR-1	**FGF18**	FGF-18
**CHRDL1**	Chordin-Like 1	**CXCR6**	CXCR6	**FGF19**	FGF-19
**CHRDL2**	Chordin-Like 2	**CYR61**	CYR61 mAb	**FGF2**	FGF Basic
**CLEC11A**	SCGF/CLEC11a mAb	**DCN**	Decorin	**FGF20**	FGF-20

**CLU**	Clusterin	**DEFB1**	BD-1	**FGF21**	FGF-21
**CMKLR1**	Chem R23	**DEFB103A**	Beta-Defensin 3 pAb	**FGF23**	FGF-23
**CNTF**	CNTF	**DEFB4A**	beta-Defensin 2	**FGF3**	FGF3 mAb
**CNTFR**	CNTF R alpha	**DKK1**	Dkk-1	**FGF4**	FGF-4
**COL18A1**	Endostatin	**DKK3**	Dkk-3	**FGF5**	FGF-5
**CR1**	CR1 (CD35)	**DKK4**	Dkk-4	**FGF6**	FGF-6
**CR2**	CR2 (CD21)	**DKKL1**	Soggy-1	**FGF7**	FGF-7 / KGF
**CRIM1**	CRIM 1	**DLK1**	Pref-1	**FGF8**	FGF-8
**CRP**	CRP	**DLL1**	DLL1 pAb	**FGF9**	FGF-9
**CSF1**	M-CSF	**EDA2R**	EDA-A2	**FGFBP1**	FGF-BP
**CSF1R**	M-CSF R	**EDAR**	EDAR	**FGFR3**	FGF R3
**CSF2**	GM-CSF	**EDN1**	Endothelin	**FGFR4**	FGF R4
**CSF2RA**	GM-CSF R alpha	**EGF**	EGF	**FGFRL1**	FGF R5
**CSF3**	GCSF	**EGFR**	EGF R / ErbB1	**FGL1**	Hepassocin
**CSF3R**	G-CSF R / CD 114	**ENG**	Endoglin / CD105	**FIGF**	VEGF-D
**CST3**	Cystatin C	**EPO**	Erythropoietin	**FLT3LG**	Flt-3 Ligand
**CTF1**	Cardiotrophin-1 / CT-1	**ERBB2**	ErbB2	**FLT4**	VEGF R3
**CTGF**	CTGF / CCN2	**ERBB3**	ErbB3	**FRZB**	sFRP-3
**CTLA4**	CTLA-4 /CD152	**ERBB4**	ErbB4	**FSHB**	FSH
**CTNNB1**	beta-Catenin	**EREG**	Epiregulin	**FST**	Follistatin
**CX3CL1**	Fractalkine	**ESM1**	Endocan	**FSTL1**	Follistatin-like 1
**CXCL1**	GRO	**F13A1**	Factor XIIIa mAb	**FSTL3**	FLRG
**CXCL10**	IP-10	**F3**	Coag. Factor III / TF	**FTL**	Ferritin mAb (heavy&light)
**CXCL11**	I-TAC / CXCL11	**F5**	Factor V pAb	**FURIN**	Furin mAb
**CXCL12**	SDF-1 / CXCL12	**FAM3B**	FAM3B	**FUT4**	CD15 mAb
**CXCL13**	BLC / BCA-1 / CXCL13	**FAS**	Fas / TNFRSF6	**FZD1**	Frizzled-1
**CXCL14**	CXCL14 / BRAK	**FASLG**	Fas Ligand	**FZD3**	Frizzled-3
**CXCL16**	CXCL16	**FBLN5**	DANCE	**FZD4**	Frizzled-4
**CXCL2**	MIP 2	**FGA**	Fibrinogen alpha	**FZD5**	Frizzled-5
**CXCL5**	ENA-78	**FGF1**	FGF acidic mAb	**FZD6**	Frizzled-6
**CXCL6**	GCP-2 / CXCL6	**FGF10**	FGF-10 / KGF-2	**FZD7**	Frizzled-7
**CXCL9**	MIG	**FGF11**	FGF-11	**GALP**	GALP
**CXCR1**	CXCR1 / IL-8 RA	**FGF12**	FGF-12	**GC**	Vitamin D binding protein mAb
**CXCR2**	CXCR2 / IL-8 RB	**FGF13**	FGF-13 1B	**GCG**	Glucagon
**GDF1**	GDF1	**IFNG**	IFN-gamma	**IL1B**	IL-1 beta
**GDF10**	BMP-3b / GDF-10	**IFNGR1**	IFN-gamma R1	**IL1F10**	IL-1 F10 / IL-1HY2
**GDF11**	GDF11	**IGF1**	IGF-I	**IL1F5**	IL-1 F5 / FIL1delta
**GDF15**	GDF-15	**IGF1R**	IGF-I SR	**IL1F6**	IL-1 F6 / FIL1 epsilon
**GDF2**	BMP-9 mAb	**IGF2**	IGF-II	**IL1F7**	IL-1 F7 / FIL1 zeta
**GDF3**	GDF3	**IGF2R**	IGF-II R	**IL1F8**	IL-1 F8 / FIL1 eta
**GDF5**	GDF5	**IGFBP1**	IGFBP-1	**IL1F9**	IL-1 F9 / IL-1 H1
**GDF6**	BMP-13 pAb	**IGFBP2**	IGFBP-2	**IL1R1**	IL-1 sRI
**GDF9**	GDF9	**IGFBP3**	IGFBP-3	**IL1R2**	IL-1 sRII
**GDNF**	GDNF	**IGFBP4**	IGFBP-4	**IL1RAP**	IL-1 R3 / IL-1 R AcP
**GFRA1**	GFR alpha-1	**IGFBP6**	IGFBP-6	**IL1RAPL1**	IL-1 R8
**GFRA2**	GFR alpha-2	**IGFBP7**	IGFBP-rp1 / IGFBP-7	**IL1RAPL2**	IL-1 R9
**GFRA3**	GFR alpha-3	**IL10**	IL-10	**IL1RL1**	IL-1 R4 /ST2
**GFRA4**	GFR alpha-4	**IL10RA**	IL-10 R alpha	**IL1RL2**	IL-1 R6 / IL-1 Rrp2

**GH1**	Growth Hormone (GH)	**IL10RB**	IL-10 R beta	**IL1RN**	IL-1 ra
**GHR**	Growth Hormone R (GHR)	**IL11**	IL-11	**IL2**	IL-2
**GHRL**	Ghrelin	**IL12A**	IL-12 p70	**IL20**	IL-20
**GP1BA**	GPIb alpha mAb	**IL12B**	IL-12 p40	**IL20RA**	IL-20 R alpha
**GPC3**	Glypican 3	**IL12RB1**	IL-12 R beta 1	**IL20RB**	IL-20 R beta
**GPC5**	Glypican 5	**IL12RB2**	IL-12 R beta 2	**IL21**	IL-21
**GPNMB**	Osteoactivin / GPNMB	**IL13**	IL-13	**IL21R**	IL-21 R
**GPR39**	Obestatin R (GPR-39)	**IL13RA1**	IL-13 R alpha 1	**IL22**	IL-22
**GPR44**	CRTH-2	**IL13RA2**	IL-13 R alpha 2	**IL22RA1**	IL-22 R
**GPX3**	Glutathione peroxidase 3	**IL15**	IL-15	**IL22RA2**	IL-22 BP
**GREM1**	GREMLIN	**IL15RA**	IL-15 R alpha	**IL23A**	IL-23
**GRN**	Progranulin pAb (0.5 mg/ml)	**IL16**	IL-16	**IL23R**	IL-23 R
**GRN**	Progranulin	**IL17A**	IL-17	**IL24**	IL-24
**GZMA**	Granzyme A	**IL17B**	IL-17B	**IL25**	IL-17E
**HBEGF**	HB-EGF	**IL17C**	IL-17C	**IL26**	IL-26
**HGF**	HGF	**IL17D**	IL-17D	**IL27**	IL-27
**HP**	Haptoglobin mAb	**IL17F**	IL-17F	**IL27RA**	TCCR / WSX-1
**HPX**	Hemopexin mAb	**IL17RA**	IL-17R	**IL28A**	IL-28A
**IAPP**	Amylin	**IL17RB**	IL-17B R	**IL29**	IL-29
**ICAM1**	ICAM-1	**IL17RC**	IL-17RC	**IL2RA**	IL-2 R alpha
**ICAM2**	ICAM-2	**IL17RD**	IL-17RD	**IL2RB**	IL-2 R beta /CD122
**ICAM5**	ICAM-5	**IL18BP**	IL-18 BPa	**IL2RG**	IL-2 R gamma
**IDE**	Insulysin / IDE	**IL18R1**	IL-18 R alpha /IL-1 R5	**IL3**	IL-3
**IFNAR1**	IFN-alpha / beta R1	**IL18RAP**	IL-18 R beta /AcPL	**IL31**	IL-31
**IFNAR2**	IFN-alpha / beta R2	**IL19**	IL-19	**IL31RA**	IL-31 RA
**IFNB1**	IFN-beta	**IL1A**	IL-1 alpha	**IL32**	IL-32 alpha mAb
**IL3RA**	IL-3 R alpha	**LIF**	LIF	**NBL1**	DAN
**IL4**	IL-4	**LIFR**	LIF R alpha	**NCAM1**	NCAM-1 / CD56
**IL4R**	IL-4 R	**LOX**	LOX	**NGF**	beta-NGF
**IL5**	IL-5	**LRP1**	LRP-1	**NGFR**	NGF R
**IL5RA**	IL-5 R alpha	**LRP6**	LRP-6	**NLGN2**	Neuroligin 2 pAb
**IL6**	IL-6	**LTA**	TNF-beta	**NLGN3**	Neuroligin 3 mAb
**IL6R**	IL-6 R	**LTB**	Lymphotoxin beta / TNFSF3	**NLGN4X**	Neuroligin 4 (X/Y) mAb
**IL6ST**	sgp130	**LTBP1**	Latent TGF-beta bp1	**NODAL**	Nodal pAb
**IL7**	IL-7	**LTBR**	Lymphotoxin beta R / TNFRSF3	**NOG**	Noggin mAb
**IL7R**	IL-7 R alpha	**MASP1**	MASP1 pAb	**NOV**	NOV / CCN3
**IL8**	IL-8	**MASP2**	MASP2 pAb	**NPY**	Neuropeptide Y pAb
**IL9**	IL-9	**MBL2**	MBL	**NRG1**	NRG1 Isoform GGF2
**INHBA**	Inhibin A	**MDK**	Midkine pAb	**NRG1**	NRG1-beta1 / HRG1-beta1
**INHBA**	Activin A	**MET**	HGFR	**NRG1**	HRG-alpha
**INHBB**	Inhibin B	**MFGE8**	MFG-E8	**NRG2**	NRG2
**INHBB**	Activin B	**MFRP**	MFRP	**NRG3**	NRG3
**INHBC**	Activin C	**MIA**	MIA mAb	**NRN1**	Neuritin
**INS**	Insulin	**MIF**	MIF	**NRP2**	Neuropilin-2
**INSL3**	INSL3	**MME**	Neprilysin pAb	**NRTN**	Neurturin
**INSR**	Insulin R	**MMP1**	MMP-1	**NRXN2**	Neurexin II alpha pAb
**INSRR**	INSRR	**MMP10**	MMP-10	**NRXN3**	Neurexin III alpha pAb
**ITGAL**	LFA-1 alpha	**MMP11**	MMP-11 /Stromelysin-3	**NTF3**	NT-3

**ITGAM**	MAC-1	**MMP12**	MMP-12	**NTF4**	NT-4
**ITGB2**	CD18	**MMP13**	MMP-13	**NTN1**	Netrin1 pAb
**JAG1**	Jagged 1 mAb	**MMP14**	MMP-14	**NTN4**	Netrin 4 mAb
**KDR**	VEGF R2 (KDR)	**MMP15**	MMP-15	**OSM**	OSM
**KIT**	SCF R /CD117	**MMP16**	MMP-16 / MT3-MMP	**OSTN**	Osteocrin
**KITLG**	SCF	**MMP19**	MMP-19	**OTOR**	Otoraplin pAb
**KNG1**	Kininostatin / kininogen	**MMP2**	MMP-2	**PDGFA**	PDGF-AA
**KREMEN1**	Kremen-1	**MMP20**	MMP-20	**PDGFA**	PDGF-AB
**KREMEN2**	Kremen-2	**MMP24**	MMP-24 / MT5-MMP	**PDGFB**	PDGF-BB
**LBP**	LBP	**MMP25**	MMP-25 / MT6-MMP	**PDGFC**	PDGF-C
**LCN1**	Lipocalin-1	**MMP3**	MMP-3	**PDGFD**	PDGF-D
**LECT2**	LECT2	**MMP7**	MMP-7	**PDGFRA**	PDGF R alpha
**LEFTY2**	Lefty - A	**MMP8**	MMP-8	**PDGFRB**	PDGF R beta
**LEP**	Leptin (OB)	**MMP9**	MMP-9	**PECAM1**	PECAM-1 /CD31
**LEPR**	Leptin R	**MST1**	MSP beta-chain	**PF4**	PF4 / CXCL4
**LGALS1**	Galectin -1	**MST1**	MSP alpha Chain	**PGF**	PlGF
**LGALS3**	Galectin-3	**MSTN**	GDF8	**PHB**	Prohibitin
**LHB**	Luteinizing Hormone	**MUSK**	Musk	**PLAU**	uPA
**PLAUR**	uPAR	**SLC2A2**	Glut2	**TNC**	Tenascin C pAb
**PLG**	Angiostatin	**SLC2A5**	Glut5	**TNF**	TNF-alpha
**PLIN2**	ADFP	**SLPI**	SLPI	**TNFAIP6**	TSG-6
**PLUNC**	PLUNC	**SORL1**	SorL1 pAb	**TNFRSF10A**	TRAILR1 / DR4 / TNFRSF10A
**POMC**	ACTH	**SOST**	SOST mAb	**TNFRSF10B**	TRAILR2 / DR5 / TNFRSF10B
**PPBP**	NAP2 pAb	**SPARC**	Osteonectin	**TNFRSF10C**	TRAILR3 / TNFRSF10C
**PRL**	Prolactin	**SPN**	ACE / CD43	**TNFRSF10D**	TRAILR4 / TNFRSF10D
**PRNP**	Prion protein Prp pAb	**SPP1**	Osteopontin mAb	**TNFRSF11A**	RANK / TNFRSF11A
**PROK1**	EG-VEGF / PK1	**SST**	Somatostatin pAb	**TNFRSF11B**	Osteoprotegerin / TNFRSF11B
**PSPN**	Persephin	**TDGF1**	Cripto-1	**TNFRSF13B**	TACI / TNFRSF13B
**PTHLH**	PTHLH pAb	**TEK**	Tie-2	**TNFRSF13C**	BAFF R / TNFRSF13C
**PTN**	Pleiotrophin pAb	**TF**	Transferrin mAb	**TNFRSF14**	HVEM / TNFRSF14
**PTX3**	Pentraxin3 / TSG-14	**TFF2**	TFF2 mAb	**TNFRSF17**	BCMA / TNFRSF17
**PYY**	PYY	**TFF3**	TFF3 mAb	**TNFRSF18**	GITR / TNFRF18
**RBP4**	RBP4	**TFPI**	TFPI	**TNFRSF19**	TROY / TNFRSF19
**RELN**	Reelin (G-20) pAb	**TGFA**	TGF-alpha	**TNFRSF1A**	TNF RI / TNFRSF1A
**RELT**	RELT / TNFRSF19L	**TGFB1**	LAP (TGF-B1)	**TNFRSF1B**	TNF RII / TNFRSF1B
**RETN**	Resistin	**TGFB1**	TGF-B1 mAb	**TNFRSF21**	DR6 / TNFRSF21
**RETNLB**	RELM beta	**TGFB2**	TGF-beta 2	**TNFRSF25**	TWEAK R / TNFRSF12
**ROBO4**	ROBO4	**TGFB3**	TGF-beta 3	**TNFRSF6B**	DcR3 / TNFRSF6B
**S1PR1**	EDG-1	**TGFBR1**	TGF-beta RI / ALK-5	**TNFRSF8**	CD30 / TNFRSF8
**SAA1**	SAA	**TGFBR2**	TGF-beta RII	**TNFRSF9**	CD137 / 4-1BB
**SDC3**	Syndecan-3	**TGFBR3**	TGF-beta RIII	**TNFSF10**	TRAIL / TNFSF10
**SELE**	E-Selectin	**THBS1**	Thrombospondin (TSP)	**TNFSF11**	TRANCE
**SELL**	L-Selectin (CD62L)	**THBS2**	Thrombospondin-2	**TNFSF12**	TWEAK / TNFSF12
**SELP**	P-selectin	**THBS4**	Thrombospondin-4	**TNFSF13**	APRIL
**SEMA3A**	SEMA3A	**THPO**	Thrombopoietin (TPO)	**TNFSF13B**	BAFF/BLyS/TNFSF13B mAb
**SERPINA1**	alpha 1 antitrypsin pAb	**TIE1**	Tie-1	**TNFSF14**	LIGHT / TNFSF14
**SERPINA12**	Vaspin	**TIMP1**	TIMP-1	**TNFSF15**	VEGI / TNFSF15
**SERPINF1**	PEDF	**TIMP2**	TIMP-2	**TNFSF18**	GITR Ligand / TNFSF18

**SERPING1**	C1Inhib	**TIMP3**	TIMP-3	**TNFSF4**	OX40 Ligand / TNFSF4
**SFRP1**	sFRP-1	**TIMP4**	TIMP-4	**TNFSF8**	CD30 Ligand / TNFSF8
**SFRP4**	sFRP-4	**TLR1**	TLR1	**TNFSF9**	4-1BB ligand/TNFSF9 mAb
**SHBG**	SHBG mAb	**TLR2**	TLR2	**TREM1**	TREM-1
**SHH**	Sonic Hedgehog (Shh N-terminal)	**TLR3**	TLR3	**TSHB**	TSH
**SIGIRR**	SIGIRR	**TLR4**	TLR4	**TSLP**	TSLP
**SIGLEC5**	Siglec-5/CD170	**TMEFF1**	TMEFF1 / Tomoregulin-1	**TYMP**	PD-ECGF
**SIGLEC9**	Siglec-9	**TMEFF2**	TMEFF2	**TYRO3**	Dtk
**SLC2A1**	Glut1	**TMPO**	Thymopoietin	**VASN**	Vasorin
**SLC2A14**	Glut3	**TMPRSS5**	Spinesin	**VCAM1**	VCAM-1 (CD106)
**VEGFA**	VEGF				
**VEGFB**	VEGF-B				
**VEGFC**	VEGF-C				
**VTN**	S Protein (Vitronectin)				
**VWF**	von Willebrand Factor A2 mAb				
**WFIKKN1**	GASP-2 / WFIKKN				
**WIF1**	WIF-1				
**WISP1**	WISP-1 / CCN4				
**WISP2**	WISP2 pAb				
**WISP3**	WISP3 pAb				
**WNT3A**	Wnt3a pAb				
**XCL1**	Lymphotactin / XCL1				

The 612 signaling proteins in alphabetic order based on their HUGO gene names and their commercial antibody names. Some proteins/genes have multiple antibodies, if antibodies exist against distinct protein modifications or precursors with potentially different biological functions (e.g. C3 or TGFB1).

Table S2: HIV infected patients used for Communicome analyisis

Group	*Age (years)*	*Gender (M/F)*	*Viral Load (plasma RNA copies/ml)*	*CD4 count (cells/mm3)*	*CTL Breadth*	*CTL Magnitude*	*Proviral*	*Communicome*	*RT-PCR*
HIV-Low 01	34	M	950	608	17	14,330	163.6	*Analyzed*	*Analyzed*
HIV-Low 02	56	F	<50	873	12	22,850	16	*Analyzed*	*Analyzed*
HIV-Low 03	41	M	<50	616	78	46,330	ND	*Analyzed*	*ND*
HIV-Low 04	35	M	9,999	438	27	24,300	317.7	*Analyzed*	*Analyzed*
HIV-Low 05	46	M	1,100	485	52	66,380	76.7	*Analyzed*	*Analyzed*
HIV-Low 06	46	F	<50	1,083	15	13,280	6.7	*Analyzed*	*Analyzed*
HIV-Low 07	40	M	210	786	11	3,820	ND	*Analyzed*	*ND*
HIV-Low 08	48	F	8,900	913	49	36,910	412.5	*Analyzed*	*Analyzed*
HIV-Low 09	28	M	1,000	889	24	11,700	ND	*Analyzed*	*ND*
HIV-Low 10	37	M	810	665	15	30,085	ND	*Analyzed*	*ND*
HIV-Low 11	37	F	1,800	511	50	32,241	180.8	*Analyzed*	*Analyzed*
HIV-Low 12	50	F	<50	487	69	45,658	9.1	*Analyzed*	*Analyzed*
HIV-Low 13	40	F	340	832	44	45,007	294.3	*Analyzed*	*Analyzed*
HIV-Low 14	37	F	<50	665	10	8,500	25.7	*Analyzed*	*Analyzed*
HIV-Low 15	38	M	160	512	29	48,160	ND	*Analyzed*	*ND*
HIV-Low 16	33	M	530	948	24	26,890	25.8	*Analyzed*	*Analyzed*
HIV-Low 17	34	M	4,300	587	14	4,640	ND	*Analyzed*	*ND*
HIV-Low 18	29	M	<50	892	9	3,220	0	*Analyzed*	*Analyzed*
HIV-Low 19	27	M	7,700	733	47	24,198	ND	*Analyzed*	*ND*
HIV-Low 20	45	F	2,900	582	63	42,517	278.5	*Analyzed*	*Analyzed*
HIV-Low 21	30	F	<50	1,114	17	8,200	ND	*Analyzed*	*ND*
HIV-Low 22	46	F	2,800	434	ND	ND	32.5	*Analyzed*	*Analyzed*
HIV-Low 23	38	M	<50	583	30	13,041	90.4	*Analyzed*	*Analyzed*
HIV-Low 24	26	M	2,300	642	27	44,150	49.1	*Analyzed*	*Analyzed*
HIV-Low 25	42	M	5,600	531	40	27,660	ND	*Analyzed*	*ND*
HIV-Low 26	44	M	50	872	35	16,229	6.2	*Analyzed*	*Analyzed*
HIV-Low 27	48	M	50	657	ND	ND	ND	*Analyzed*	*ND*
HIV-Low 28	41	F	<50	672	7	1,530	20.9	*Analyzed*	*Analyzed*
HIV-Low 29	50	F	50	1,228	22	25,600	0	*Analyzed*	*Analyzed*
HIV-Low 30	46	M	66	755	ND	ND	28.6	*Analyzed*	*Analyzed*
HIV-Low 31	51	M	<50	450	ND	ND	41.5	*Analyzed*	*Analyzed*
HIV-Low 32	48	F	230	516	39	15,810	15.9	*Analyzed*	*Analyzed*
HIV-Low 33	43	M	330	1,237	15	5,490	31.3	*Analyzed*	*Analyzed*
HIV-Low 34	40	F	1,500	1,084	ND	ND	ND	*Analyzed*	*ND*
HIV-Low 35	48	M	50	601	ND	ND	18.4	*Analyzed*	*Analyzed*
HIV-Low 36	35	F	<50	930	5	660	11.5	*Analyzed*	*Analyzed*
HIV-Low 37	36	F	880	1,343	9	2,630	12.1	*Analyzed*	*Analyzed*
HIV-Low 38	40	F	4,800	569	16	6,580	153.5	*Analyzed*	*ND*
HIV-Low 39	57	F	50	485	10	5,390	ND	*Analyzed*	*ND*
HIV-Low 40	45,8	M	1,700	742	ND	ND	ND	*Analyzed*	*ND*
HIV-Low 41	48	M	<50	593	ND	ND	ND	*Analyzed*	*ND*
HIV-Low 42	31	M	9,756	404	10	1,850	620.7	*Analyzed*	*ND*
HIV-Low 43	29	M	5,624	823	29	8,170	1,662.4	*Analyzed*	*Analyzed*
HIV-Low 45	41	M	6,127	531	14	2,300	50.2	*Analyzed*	*Analyzed*
HIV-Low 46	34	M	400	1,035	4	730	220.1	*Analyzed*	*Analyzed*
HIV-Low 47	33	F	7,923	524	2	480	366.5	*Analyzed*	*Analyzed*
HIV-Low 48	30	F	8,858	289	4	1,780	ND	*Analyzed*	*ND*
HIV-Low 49	39	F	4,424	419	16	4,290	ND	*Analyzed*	*ND*
HIV-Low 50	30	M	4,482	1,151	10	2,610	0	*Analyzed*	*Analyzed*
HIV-High 01	21	F	269,431	212	8	1,150	4.2	*Analyzed*	*ND*
HIV-High 02	28	F	74,922	571	4	280	ND	*Analyzed*	*ND*
HIV-High 03	27	F	108,103	369	13	6,240	ND	*Analyzed*	*ND*
HIV-High 06	37	F	140,000	11	15	19,650	231.9	*Analyzed*	*Analyzed*
HIV-High 07	40	F	64,000	282	12	4,415	922.6	*Analyzed*	*Analyzed*
HIV-High 08	45	F	86,000	90	50	49,480	ND	*Analyzed*	*ND*
HIV-High 09	24	F	610,000	128	15	4,428	1,722.4	*Analyzed*	*Analyzed*
HIV-High 10	47	F	510,000	33	18	8,400	ND	*Analyzed*	*ND*
HIV-High 11	33	F	1,200,000	75	15	20,050	80.1	*Analyzed*	*Analyzed*
HIV-High 12	50	F	54,000	67	7	2,040	363.6	*Analyzed*	*Analyzed*
HIV-High 13	42	F	1,100,000	283	21	41,420	ND	*Analyzed*	*ND*
HIV-High 14	45	F	68,000	98	12	9,420	ND	*Analyzed*	*ND*
HIV-High 15	44	F	510,000	12	10	2,100	ND	*Analyzed*	*ND*
HIV-High 16	21	M	80,328	437	23	19,940	ND	*Analyzed*	*ND*
HIV-High 17	22	M	152,554	293	8	1,340	3,143.4	*Analyzed*	*ND*
HIV-High 18	22	M	53,299	356	14	3,450	ND	*Analyzed*	*ND*
HIV-High 19	23	M	70,393	208	10	11,530	223.7	*Analyzed*	*ND*
HIV-High 20	23	M	55,733	340	4	1,320	ND	*Analyzed*	*ND*
HIV-High 21	23	M	50,295	505	7	3,910	1,176.5	*Analyzed*	*Analyzed*
HIV-High 22	23	M	240,039	388	ND	ND	1,144,6	*Analyzed*	*Analyzed*
HIV-High 23	24	M	93,928	256	1	50	3,246,6	*Analyzed*	*Analyzed*
HIV-High 24	24	M	365,977	726	35	18,535	1,204,6	*Analyzed*	*Analyzed*
HIV-High 25	24	M	150,176	407	10	2,434	ND	*Analyzed*	*ND*
HIV-High 26	25	M	60,467	634	16	8,080	ND	*Analyzed*	*ND*
HIV-High 27	26	M	750,000	239	28	9,900	941.4	*Analyzed*	*ND*

HIV-High 28	27	M	54,304	704	20	102,00	ND	*Analyzed*	*ND*
HIV-High 29	28	M	216,105	313	15	3,480	3,021.1	*Analyzed*	*ND*
HIV-High 30	28	M	327,087	544	10	5,790	2,127.2	*Analyzed*	*Analyzed*
HIV-High 31	29	M	418,022	253	15	4,790	ND	*Analyzed*	*ND*
HIV-High 32	29	M	125,701	404	9	6,320	ND	*Analyzed*	*ND*
HIV-High 33	30	M	>750,000	244	7	1,980	ND	*Analyzed*	*ND*
HIV-High 34	30	M	61,078	380	22	27,750	558.4	*Analyzed*	*Analyzed*
HIV-High 35	31	M	194,091	271	7	590	1,962.5	*Analyzed*	*Analyzed*
HIV-High 36	31	M	85,102	365	11	5,350	ND	*Analyzed*	*ND*
HIV-High 38	32	M	57,021	480	14	16,140	ND	*Analyzed*	*ND*
HIV-High 39	32	M	170,000	17	83	145,250	507	*Analyzed*	*Analyzed*
HIV-High 40	33	M	320,000	192	51	21,200	ND	*Analyzed*	*ND*
HIV-High 41	33	M	97,483	451	21	36,010	ND	*Analyzed*	*ND*
HIV-High 42	35	M	71,739	227	5	690	1,562.5	*Analyzed*	*ND*
HIV-High 43	35	M	95,365	240	5	660	3,149.2	*Analyzed*	*Analyzed*
HIV-High 44	35	M	102,936	283	35	12,790	1,346.3	*Analyzed*	*Analyzed*
HIV-High 45	35	M	63,381	384	44	46,800	1,128.7	*Analyzed*	*Analyzed*
HIV-High 46	36	M	126,557	364	23	32,690	ND	*Analyzed*	*ND*
HIV-High 47	37	M	61,000	80	0	0	ND	*Analyzed*	*ND*
HIV-High 48	37	M	61,011	641	3	930	ND	*Analyzed*	*ND*
HIV-High 49	37	M	140,000	20	35	7,060	ND	*Analyzed*	*ND*
HIV-High 50	38	M	544,275	402	33	22,080	ND	*Analyzed*	*ND*

ND: Not determined
CTL_B: Number of reactive peptides
CTL_M: Median of SFC per 10^6 PBMC
M: Male
F: Female

Table S3: Independent cohorts used for ELISA and RT-PCR validation

	Group	*Age (years)*	*Gender (M/F)*	*Viral Load (RNA copies/ml)*	*CD4 count (cells/mm3)*	*Proviral*	*Time pre HIV innfection and pre or post (cART treatment) (days)*	*ELISA*	*RT-PCR*
**Seronegatives**	Seroneg_01	35	M	NA	NA	ND	-120	Analyzed	ND
	Seroneg_02	30	M	NA	NA	1245.8	-60	Analyzed	Analyzed
	Seroneg_03	21	M	NA	NA	4.8	-60	Analyzed	Analyzed
	Seroneg_04	24	M	NA	NA	ND	-60	Analyzed	ND
	Seroneg_05	34	M	NA	NA	384.2	-90	Analyzed	Analyzed
	Seroneg_06	33	M	NA	NA	5.9	-60	Analyzed	Analyzed
	Seroneg_07	32	M	NA	NA	16.9	-60	Analyzed	Analyzed
	Seroneg_08	27	M	NA	NA	0	-60	Analyzed	Analyzed
**Chronics HIV infected**	Chr-Untreat-5	46	M	4,900	462	175.5	-12	Analyzed	Analyzed
	Chr-Untreat-6	40	M	15,000	504	281.6	-11	Analyzed	Analyzed
	Chr-Untreat-11	28	F	15,000	456	549.8	-146	Analyzed	Analyzed
	Chr-Untreat-12	44	M	50,000	629	222.2	-90	Analyzed	Analyzed
	Chr-Untreat-14	29	M	13,284	661	8.5	-49	Analyzed	ND
	Chr-Untreat-15	37	M	8,110	432	317.5	-36	Analyzed	Analyzed
	Chr-treat-5	47	M	<50	532	52.3	329	Analyzed	Analyzed
	Chr-treat-9	33	M	<25	460	387.5	367	Analyzed	Analyzed
	Chr-treat-11	30	F	<25	786	191.4	480	Analyzed	Analyzed
	Chr-treat-12	45	M	<50	558	43	367	Analyzed	Analyzed
	Chr-treat-13	36	F	<40	720	115.9	347	Analyzed	Analyzed
	Chr-treat-15	38	M	<40	659	89.3	474	Analyzed	Analyzed
**Chronics HIV infected Controllers**	VC_01	45	F	790	588	466.6	N.A	Analyzed	Analyzed
	VC_02	40	M	55	1,014	27.1	N.A	Analyzed	Analyzed
	VC_03	48	M	1,978	1,840	9.4	N.A	Analyzed	Analyzed
	VC_04	36	F	374	594	74.9	N.A	Analyzed	Analyzed
	VC_05	38	M	972	405	38.2	N.A	Analyzed	Analyzed
	VC_06	44	F	1,300	510	64.4	N.A	Analyzed	Analyzed
	VC_07	47	M	1,200	680	35.4	N.A	Analyzed	Analyzed
	VC_08	47	M	1,500	538	324.9	N.A	Analyzed	Analyzed
	VC_09	41	M	200	603	56.5	N.A	Analyzed	Analyzed
	VC_10	54	M	480	540	75.7	N.A	Analyzed	Analyzed
	VC_11	52	F	1,600	448	201.8	N.A	Analyzed	Analyzed
	EC_01	31	F	<25	922	0	N.A	Analyzed	Analyzed
	EC_03	40	F	<40	752	8.5	N.A	Analyzed	Analyzed
	EC_04	46	F	<25	1,557	5.6	N.A	Analyzed	Analyzed
	EC_05	43	F	<25	372	4.3	N.A	Analyzed	Analyzed
	EC_06	59	M	<25	411	11.1	N.A	Analyzed	Analyzed
	EC_07	40	F	<25	245	180.2	N.A	Analyzed	Analyzed
	EC_09	40	F	56	898	3.8	N.A	Analyzed	Analyzed
	EC_10	50	M	<25	450	0	N.A	Analyzed	Analyzed
	EC_12	46	M	<25	940	3.4	N.A	Analyzed	Analyzed
	EC_13	52	M	<25	667	0	N.A	Analyzed	Analyzed
	EC_15	53	M	<25	959	20.8	N.A	Analyzed	Analyzed
	EC_18	55	F	<50	838	23.8	N.A	Analyzed	Analyzed

ND: Not determined
M: Male
F: Female

Figure S1.

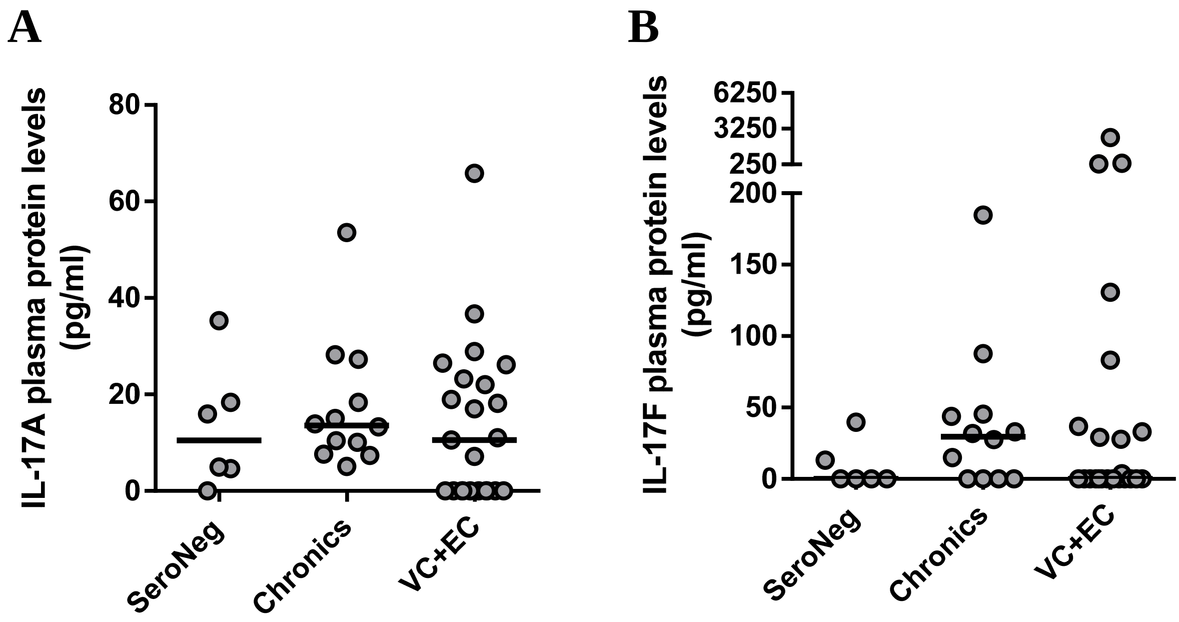

Figure S1: IL-17A/F plasma levels during HIV infection. A) Differential plasma levels of IL-17A and B) IL-17F detected by Elisa in validation cohorts including HIV seronegatives (n=8), chronically infected HIV infected individuals (n=12) and controllers subjects (n=23). Mann Whitney test was applied for groups comparisons and p-values < 0.05 were consider significant
